# Depression, Anxiety, and Apathy in Mild Cognitive Impairment: Current Perspectives

**DOI:** 10.3389/fnagi.2020.00009

**Published:** 2020-01-30

**Authors:** Lina Ma

**Affiliations:** Department of Geriatrics, Xuanwu Hospital, Capital Medical University, China National Clinical Research Center for Geriatric Medicine, Beijing, China

**Keywords:** mild cognitive impairment, depression, anxiety, apathy, dementia

## Abstract

**Objective**: Mild cognitive impairment (MCI) is an important risk state for dementia, particularly Alzheimer’s disease (AD). Depression, anxiety, and apathy are commonly observed neuropsychiatric features in MCI, which have been linked to cognitive and functional decline in daily activities, as well as disease progression. Accordingly, the study’s objective is to review the prevalence, neuropsychological characteristics, and conversion rates to dementia between MCI patients with and without depression, anxiety, and apathy.

**Methods**: A PubMed search and critical review were performed relating to studies of MCI, depression, anxiety, and apathy.

**Results**: MCI patients have a high prevalence of depression/anxiety/apathy; furthermore, patients with MCI and concomitant depression/anxiety/apathy have more pronounced cognitive deficits and progress more often to dementia than MCI patients without depression/anxiety/apathy.

**Conclusions and Implications**: Depression, anxiety, and apathy are common in MCI and represent possible risk factors for cognitive decline and progression to dementia. Further studies are needed to better understand the role and neurobiology of depression, anxiety, and apathy in MCI.

## Introduction

Mild cognitive impairment (MCI) represents a transitional stage between healthy aging and dementia. Subjects with MCI complain about cognitive impairments, have documented cognitive deficits relative to age- and education-matched controls—although they are less impaired than patients with dementia—and have largely intact activities of daily living (Sanford, 2017). Depending on the inclusion criteria, the prevalence of MCI in the general older population has been estimated between 5.0%–36.7% (Sachdev et al., 2015) and 11%–33% of subjects with MCI develop dementia within 2 years (Luis et al., 2003; Bruscoli and Lovestone, 2004). MCI is thus regarded as an important risk state for dementia.

However, longitudinal studies showed that up to 50% of patients with MCI at their first doctor visit returned to normal at follow-up examinations (Larrieu et al., 2002; Ganguli et al., 2004), particularly in population-based cohorts. Apart from neurodegenerative disorders, many factors affect cognitive performance in older populations, including age, gender, education, vascular risk factors, genetic background, hormonal changes, and comorbidities (Yaffe, 2018). Additionally, studies of MCI differed greatly as to their inclusion criteria, study settings, sample characteristics, classification and subtype of MCI, observation period, and evaluation of cognitive and other relevant features. Combined with the biological heterogeneity of the MCI syndrome, these factors are the reason why the prognosis of cognitive deficits in the older adults and in MCI varies greatly across studies. Furthermore, a lot of MCI patients revert to a normal stage. This could be due to lack of biomarkers at the time of the diagnoses, which makes it difficult to draw inferences on the nature of psychiatric symptoms. Alzheimer’s disease (AD) is defined by its underlying pathologic processes that can be documented by postmortem examination or *in vivo* by biomarkers in the National Institute on Aging and Alzheimer’s Association Research Framework in Jack et al. (2018). In the new framework, the diagnosis of AD is not based on the symptoms or signs but a biological construct.

## Search Criteria

We included original studies from tertiary referrals and population-based studies written in English that examined populations with MCI, depression, anxiety and apathy. A systematic literature search was performed using the PubMed database. Search terms were *depression, apathy, anxiety, neuropsychology, cognitive, conversion, progression, or prognosis* in combination with *MCI or CIND*. Articles were included if they had primary data derived from cross-sectional or longitudinal studies and were published prior to May 2018. Studies was excluded if they: (a) presented a reanalysis of subpopulations already included in other studies; (b) reported a patient population of less than 10 patients; or (c) were commentaries, technical notes, or review articles summarizing the results of previous studies.

### Psychological Symptoms in MCI

Behavioral disorders and psychological symptoms often accompany MCI and were reported to affect its presentation and course. Psychopathology, such as depression, anxiety, or apathy, are frequent in pre-dementia stages, dementia, and also in normal aging; prevalence rates for depression in normal older subjects range between 14.6% and 53% (Wada et al., 2004; Wen et al., 2010; Zivin et al., 2010; Vadla et al., 2013; Qadir et al., 2014), for anxiety between 3.7% and 43% (Bryant et al., 2008; Vadla et al., 2013; Arbus et al., 2014; Katzman et al., 2014), and for apathy between 2% and 75.2% (Adams, 2001; Lyketsos et al., 2002; Onyike et al., 2007). Cognitive impairment in major depression is well documented and is part of depression and its DSM criteria; the deterioration of processing speed and executive, attentional, and amnestic functions are frequent findings (Christensen et al., 1997; Lee R. S. C. et al., 2012; Rock et al., 2014). Cognitive deficits were found more often in older than younger depressed adults (Thomas et al., 2009; van den Kommer et al., 2012) and are more pronounced if depression was combined with anxiety (Basso et al., 2007; Rosenberg et al., 2011). Moreover, several studies have identified depression as a risk factor for disease progression and the development of dementia (Moon et al., 2017; Sugarman et al., 2018). Depressive symptoms may affect balance in MCI patients, potentially increasing the risk of falls (Pieruccini-Faria et al., 2018). Apathy, characterized by reduced motivation, reduced goal-directed behavior, and a flattening of affect (Steffens et al., 2000; Delrieu et al., 2015), may overlap with, but can be differentiated from, depression. Apathy also deteriorates cognitive functions in patients with normal aging, MCI, and dementia (Brodaty et al., 2010; Delrieu et al., 2015) and is a potential risk factor for dementia (Palmer et al., 2010; Richard et al., 2012). Similarly, anxiety—defined as anxious behavior or abnormal fear—had a reported impact on conversion from MCI to AD, directly or indirectly *via* depression (Palmer et al., 2007; Potvin et al., 2011).

In sum, depression, anxiety, and apathy are common in MCI, and a conjoint influence of psychopathology and MCI on cognitive abilities and the development of dementia has been reported (Palmer et al., 2010; Delrieu et al., 2015). However, studies of MCI differed grossly as to their methodology, such as patient selection, diagnostic criteria, assessment, and other features. Thus, findings regarding the neuropsychological and prognostic features of apathy and depression in MCI were inconsistent, and the overall picture is therefore cloudy. It also remains unclear which subtypes or characteristics of geriatric depression deteriorate cognition (Dillon et al., 2014).

### MCI Patients Have Higher Rates of Depression

The reported prevalence of depression in MCI patients ranged between 16.9%–55%, whereas only 11%–30% of older adults presented significant depressive symptoms; (Kivelä et al., 1988; Gallo and Lebowitz, 1999; Steffens et al., 2000; Copeland et al., 2004; Lee and Shinkai, 2005) this indicates that MCI patients had higher rates of depression than normal adults, and those rates were independent from age, race, gender, and study type. The different MCI definitions, depression instruments, and criteria lead to the wide range of prevalence of depression in MCI patients. A meta-analysis showed the prevalence of depression in patients with MCI is 32% (Ismail et al., 2017), but the use of anti-depressive drugs was not shown to be a protective factor of dementia (Chan et al., 2019). Other studies have shown that subjects with depression have a higher incidence of MCI (Muller et al., 2007; Ng et al., 2009). Depressive patients have more amyloid abnormalities than non-depressive patients (Donovan et al., 2018) MCI with Aβ burden of the brain is associated with an increased risk of having neuropsychiatric symptoms (Krell-Roesch et al., 2019). Individuals with MCI are at an increased risk of progression to more severe cognitive impairment (Mitchell and Shiri-Feshki, 2009) and can have subtle impairments in everyday functioning (Hughes et al., 2012) and co-occurring depressive symptoms (Byers and Yaffe, 2011). A study found the prevalence of depression in AD and multidomain-MCI was 49.6% and 44.1%, respectively (Di Iulio et al., 2010). There is a high prevalence of neuropsychiatric disturbances in patients with MCI, including depression, apathy, anxiety, aggression, and agitation (Apostolova and Cummings, 2008; Monastero et al., 2009). Neuropsychiatric symptoms containing depression and apathy as well as subjective cognitive decline may be among the symptoms of preclinical stages of AD (Rosenberg et al., 2013; Vogel et al., 2017; Jessen, 2019), and they are early manifestations of AD symptomatology and possible predictors of progression from MCI to dementia (Palmer et al., 2007, 2010; Monastero et al., 2009; Kim et al., 2013).

Depressive symptoms were commonly associated with MCI, and anxiety-depression was found to be a significant risk factor (Rodríguez-Sánchez et al., 2011; Juarez-Cedillo et al., 2012; Moretti et al., 2013). The reported prevalence of depression in MCI patients is sensitive to the criteria used to diagnose MCI and its subtypes (Sasaki et al., 2009). Subjects with MCI are more likely to develop depression compared with those with normal cognitive function, especially with those with amnestic MCI (aMCI; Hidaka et al., 2012; Shahnawaz et al., 2013). However, some studies found that there is no difference in rates of depression between aMCI and non-aMCI groups (Brown et al., 2014). MCI patients with depressive symptoms showed more severe behavioral symptoms and verbally agitated behavior (Van der Mussele et al., 2013), and some studies reported an increased AD risk in MCI patients with depression (Modrego and Ferrández, 2004), whereas other studies reported no effect (Rozzini et al., 2005; Palmer et al., 2007).

### MCI Patients With Depression Have More Cognitive Deficits

Patients with depression and MCI scored worse in memory function (Modrego and Ferrández, 2004; Brunet et al., 2011; Yoon et al., 2017), and the executive function, dementia screening, flexibility, and lexico-semantic function were significantly worse in patients with MCI with stable depression than MCI patients without depression (Lee G. J. et al., 2012). Though not every study used the same instruments, the results were consistent. The cognitive function of patients with both MCI and depression was worse than patients without depression in most studies, but there was no difference in visuoconstructional abilities, visuoperceptual abilities, and results on the Boston Naming Test in some studies between patients with MCI and those without MCI (Brunet et al., 2011; Steenland et al., 2012).

Depressed individuals tend to have lower processing speeds; (Nebes et al., 2000; Sheline et al., 2006) exhibit worse performances during tasks involving selective attention, response inhibition, and performance monitoring; and lower acquisition and retrieval of new information than non-depressed individuals (Beats et al., 1996). Late-life depression is related to deficits in short-term memory. In older subjects without dementia, depressive symptoms are associated with memory complaints (Zandi, 2004) and worse cognitive performance (Sheline et al., 2006), such as executive functions (Elderkin-Thompson et al., 2006; Sheline et al., 2006), attention, and processing speed (Elderkin-Thompson et al., 2006). MCI patients with depression also have significantly lower scores on immediate memory and delayed memory indices than MCI patients without depression (Johnson et al., 2013).

Depressive symptomatology might precede the development of cognitive decline by a decade or more (Geda et al., 2004) and is a clinical correlate of memory awareness in patients with AD dementia (O’Connell et al., 2014). Depression exacerbates pre-existing cognitive impairment by depleting cognitive reserve or otherwise lowering the threshold for the clinical manifestation of dementia (Jorm, 2001). Neuropsychiatric syndromes of apathy and depression may represent earlier signs of neurodegeneration than cognitive or functional impairments, and these behavioral prodromes may also predict different cognitive and functional trajectories (Zahodne and Tremont, 2013). Semantic deficits in aMCI are somewhat associated with the presence of concomitant depressive symptoms, but depression alone cannot account solely for the semantic deficits (Brunet et al., 2011). The presence of functional impairment was excluded in early definitions of MCI, but some recent studies have reported varying degrees of functional impairment associated with MCI (Farias et al., 2006). Furthermore, functional impairment is a defining feature of MCI and is partially dependent on the degree of cognitive impairment, and functional ability seems to be more related to depression (Bombin et al., 2012). Executive functions are independently related to anxiety disorders in MCI patients (Rozzini et al., 2009).

### MCI Patients With Depression Have Higher Conversion Rates to Dementia

The reported annual conversion rate of MCI to dementia was between 25% and 28% in the population of MCI patients with depression; MCI patients with stable depression demonstrated a significantly higher rate of conversion to AD (31%) compared to MCI patients without depression (13.5%; Lee G. J. et al., 2012). The results of a log-rank test were consistent with those findings (Modrego and Ferrández, 2004). Previous studies have found that the annual conversion rate from MCI to dementia is 4.2% in the general population and 10%–15% in high-risk clinical samples (Mitchell and Shiri-Feshki, 2009; Petersen et al., 2009). Depression is a major risk factor for incidence of dementia and is associated with greater atrophy in AD-affected regions; thus depression in individuals with MCI may be associated with underlying neuropathological changes, and depression may be a potentially useful clinical marker for identifying MCI patients who are most likely to progress to AD (Lee G. J. et al., 2012). Late-life depression is a strong risk factor for normal subjects progressing to MCI. The “always depressed” have only a modest increased risk of progression from MCI to AD, but there is no effect of prior depression (Steenland et al., 2012). A Geriatric Depression Scale (GDS) score of 6 or higher is independently associated with a much greater likelihood of developing MCI after adjusting for age, education, alcohol use, benzodiazepine use, and study site, which indicates that elevated depressive symptoms are an important risk factor for cognitive disorders and lower cognitive performance among women living to their ninth and tenth decades (Spira et al., 2012). The association between depression and cognition decline is more pronounced in MCI than AD (Lee et al., 2019). Disease progression in AD can be measured in different ways such as everyday cognition and instrumental activities of daily living (IADL; Weintraub et al., 2018). However, different types of neuropsychiatric symptoms predict different measures of AD disease progression, e.g., Affective syndromes characterized by depressive symptoms are associated with faster functional decline whereas Manic syndromes are better at predicting cognitive decline (Palmer et al., 2011). Individuals with greater symptoms of the hyperactivity and mood items on the Neuropsychiatric Inventory-Questionnaire (NPI-Q) and the presence of depressive symptoms in patients with amyloid-positive MCI are more associated with progression to AD dementia (Moon et al., 2017; Sugarman et al., 2018). A recent meta-analysis showed depressive symptoms in MCI predicted dementia in community-based studies (RR = 1.69;Tan et al., 2019).

However, there is research that demonstrated contrary conclusions. A 3-year prospective study of MCI outpatients demonstrated no increased risk of AD in patients with symptoms of depression (Palmer et al., 2010). One study found a strong negative influence of depression on conversion to AD in MCI patients (Defrancesco et al., 2017), while another study showed that depressive symptoms are not associated with the rate of progression to dementia in MCI patients and that gender moderated the association between depressive symptoms and conversion to dementia (Panza et al., 2008). Other studies found that the increased endorsement of memory problems is the only significant predictor of conversion to dementia, which likely represents insight into cognitive problems more than depressive symptomatology in MCI individuals (Mackin et al., 2012). The different results about depression in MCI may be because studies did not account for length of depression, incident depression, if it was treated, etc., since depression is not a stable state and the clinical characteristics of the depression may play a role. One study found that persistent or incident depression worsens cognitive outcome while no or recovered depression does not affect it in early AD patients (Spalletta et al., 2012). Further studies on the type and clinical characteristics of depression in MCI patients are needed.

Vascular factors play an important role in depression within preclinical dementia. In MCI patients, new onset of depression was associated with deep subcortical cerebral white matter hyperintensity severity (Kim et al., 2016). Another study showed that the cognitive decline was associated with vascular burden (white matter hyperintensity) in remitted geriatric depression patients but neurodegeneration (left hippocampal volume) in aMCI patients (Ye et al., 2017). A cohort with 35,791 participants that were followed up for 13 years showed that individuals with depression had a higher risk of dementia, and that, furthermore, depression had a more significant effect on participants with incident stroke or newly diagnosed hypertension, which indicated that targeting vascular disorders might lower dementia risk (Köhler et al., 2015). Magnetic resonance imaging (MRI) signs are independent risk factors for dementia and MCI; in a cohort study of 1,553 participants, vascular changes (subcortical microhemorrhages and infarcts) were more important in the development of MCI than in its progression to dementia, while AD signature region volume was important in both stages (Wu et al., 2019).

### Apathy: A More Important Indicator

The reported prevalence of apathy in MCI is between 10.7% and 44.8% (Palmer et al., 2010; Chan et al., 2011; Richard et al., 2012; Pink et al., 2015). Studies report differences in apathy prevalence depending on MCI type: 6.9% in amnestic-MCI and 14.7% in multidomain-MCI (Di Iulio et al., 2010). Apathy is defined as diminished motivation for at least 4 weeks and accompanied by any two of the following: reduced goal-directed behavior, reduced goal-directed cognitive activity, and reduced emotions (Robert et al., 2009). Apathy may be more likely to occur in those with frontal lobe deficits (Brodaty et al., 2005). Increased apathy is found to mediate the relationship between cognition and depression (Funes et al., 2018). Apathy is the most common and persistent neuropsychiatric symptom in AD patients, occurring in 55% of dementia patients (Aalten et al., 2007), which is a prevalent neuropsychiatric manifestation in individuals with AD. It can exert a greater impact on daily functioning than depression, which increases reliance on caregivers (Zahodne and Tremont, 2013).

Patients with MCI with apathy have an increased risk of dementia, independent of depression. A systematic review found that apathy was associated with an approximately two-fold increased risk of dementia in memory clinic patients (van Dalen et al., 2018). Robert et al. (2006) found that MCI patients with apathy develop AD more than MCI patients without apathy. After adjusting for the baseline diagnosis of apathy, the risk of progressing to AD in MCI patients with apathy is more than several times higher than in MCI patients without apathy, while there is no increased risk of progressing to AD for MCI patients with depression compared to MCI patients without depression (Palmer et al., 2010). Furthermore, Robert et al. (2008) subdivided the symptoms of apathy and found that the risk of conversion to AD was significantly higher for patients presenting a lack of interest, even after using Cox’s analyses that controls for age, gender, and education. To determine whether apathy is a more important indicator than depression and anxiety for converting MCI to dementia, further studies are needed.

### Anxiety: Another Important Symptom

Anxiety symptoms have been studied less than depression, and the relationship between anxiety and cognition is complex. The reported prevalence of anxiety in MCI patients ranged between 9.9%–52% (Lyketsos et al., 2002; Palmer et al., 2007; Chan et al., 2011; Gallagher et al., 2011). A meta-analysis showed that the prevalence of anxiety in patients with MCI was 14.3% in community-wide samples and was 31.2% in clinic-based samples (Chen et al., 2018). Such inconsistencies in reported prevalence may be attributed to differences in recruitment strategies and methodology (Gallagher et al., 2011). There is a high rate of comorbid depressive disorders in MCI patients with anxiety, confirming a positive correlation between the two neuropsychiatric disturbances in both demented and non-demented older people (Porter et al., 2003; Rozzini et al., 2009). Anxiety symptoms have been found to have a strict interaction with executive functions in MCI, and thus they may be a marker of incipient cognitive decline in MCI (Rozzini et al., 2009).

Generalized anxiety disorder is the main anxiety disorder associated with poor global cognitive functioning, and this association is moderated by sex but not by the presence of depressive episodes (Potvin et al., 2011). Anxiety symptoms are a risk factor for AD in the older adults with MCI in population-based samples (Palmer et al., 2007) but not in clinical samples (Devier et al., 2009). Biringer et al. (2005) observed that a high anxiety level was related to cognitive functioning only when it occurred with depressive symptoms, whereas Paterniti et al. (1999) found that a high anxiety level was associated with poor global cognitive functioning in non-depressed men. Some results demonstrated that high anxiety levels were negatively associated with cognitive performance (Bierman et al., 2005), while others suggested that co-morbid depressive symptoms accounted for this association (Biringer et al., 2005). Anxiety symptoms improved the predictive validity of MCI for identifying future AD, suggesting that mood-related depressive symptoms in preclinical AD may be related to the neuropathologic mechanism (Palmer et al., 2007).

Anticipatory anxiety is significantly associated with earlier conversion to AD, but this association does not remain significant following an adjustment for cognitive status at the baseline; anxiety for upcoming events and purposeless activity frequently co-occur, which indicates anticipatory anxiety may be a marker of severity rather than an independent predictor of disease progression (Gallagher et al., 2011). Devier showed that different risk profiles have been described for state vs. trait anxiety: state anxiety was not a significant predictor of future conversion to AD, but higher trait anxiety predicted a lower risk of future conversion to AD (Devier et al., 2009).

Other investigators have failed to find an association between anxiety symptoms in patients with MCI and an increased risk of conversion to AD (Robert et al., 2008; Devier et al., 2009). Anxiety level is not predictive of cognitive performance on four assessments in a 9-years follow-up (Bierman et al., 2008). and the cross-sectional association between a high level of anxiety and poor cognitive functioning is temporary (Bierman et al., 2005, 2008). Further research with long-term follow-up in larger samples is needed to clarify the role of anxiety in predicting MCI conversion to AD.

## Conclusions and Future Perspectives

Depression, anxiety, and apathy are common in MCI patients and are important indicators in the progression to dementia in MCI patients, which emphasizes the importance of assessing depressive symptoms as well as anxiety and apathy in the early stages of cognitive impairment. Further studies are needed to better understand the role and neurobiology of depression, anxiety, and apathy in MCI. Indeed, further studies on observation of larger patient populations and long follow-up are needed.

## Author Contributions

LM designed and wrote the manuscript.

## Conflict of Interest

The author declares that the research was conducted in the absence of any commercial or financial relationships that could be construed as a potential conflict of interest.
